# Supplementary material for: Are carbon emissions trading and green financial instruments synergistic? -Comprehensive quantitative research based on content analysis
Source: PLoS One. 2024 Mar 7;19(3):e0298601. doi: 10.1371/journal.pone.0298601 (PMC10919601; doi:10.1371/journal.pone.0298601)
Supplement: S1 Data — (ZIP) [file pone.0298601.s001.zip › Data/地级市Z_F工作报告.zip!/╔╜╢1⁄2/═■║ú╩╨/└┤╫╘╣1⁄2╓┌║┼í╛┬φ┐╦╩2╛▌═°í┐_2018.docx]

各位代表：

　　现在，我代表威海市人民政府向大会报告工作，请予审议，并请市政协各位委员提出意见。

　　一、2017年政府工作回顾

　　过去的一年，在省委、省政府和市委的坚强领导下，市政府和全市人民一道，深入学习贯彻习近平新时代中国特色社会主义思想，牢牢把握争当全省走在前列排头兵的目标定位，加快实施全域城市化、市域一体化和产业强市、工业带动、突破发展服务业战略，统筹做好稳增长、促改革、调结构、惠民生、防风险等各项工作，现代化幸福威海建设迈出新步伐、取得新成绩。

　　——经济运行稳中向好、质量提高。全市生产总值预计3550亿元，增长8.2%左右，比上年加快0.2个百分点；经济质量效益指标增幅总体快于生产指标，一般公共预算收入273.1亿元，增长9.5%，主要经济指标增幅保持全省第一方阵。

　　——转型升级步伐加快、持续见效。三次产业比重预计由7.0:46.4:46.6调整为6.6:45.8:47.6，高新技术产业产值比重由40%提高到41%；“三去一降一补”任务有效落实，商品住房去化周期降至16个月的合理区间。

　　——环境治理聚焦发力、成效明显。万元生产总值能耗预计降低4.9%左右，主要污染物排放量完成削减任务，PM2.5平均浓度近五年来最低，环境空气质量稳定达到国家二级标准，入选全国节水型社会创新试点示范市。

　　——民生保障全面改善、持续加强。城镇和农村居民人均可支配收入预计分别增长8.5%、7.8%左右，城镇登记失业率为1.65%，180个省定扶贫工作重点村全部“摘帽”。

　　——文明创建持续深入、再结硕果。获评全国未成年人思想道德建设工作先进城市，荣成市、乳山市成功创建第五届全国文明城市，实现全国文明城市“全域一片红”。

　　一年来，我们坚持目标导向和问题导向，突出重点、突破难点，下大气力抓了以下六个方面的工作：

　　（一）超前谋划、精准定位，大力实施新旧动能转换重大工程。坚持以供给侧结构性改革为主线，按照“四个城市”发展定位和“五区九基地”发展格局，扎实推进新旧动能转换重大事项。

　　重点产业培育取得新突破。大力推进制造业转型升级，新增国家级制造业单项冠军企业3家，战略性新兴产业主营业务收入增长14%以上。成功举办“健康中国与助推山东康养产业发展”座谈会，加快建设国际医疗健康产业城，医疗健康、养老养生等多业态融合发展。举办中国服装设计大赛、第九届中国威海国际人居节及国际建筑设计大奖赛，时尚创意产业加快破题。全域旅游扎实推进，入选全国森林旅游示范市，华夏城被评为国家5A级旅游景区。建成15个文化产业公共服务平台，规模以上文化企业数量增长57.6%。现代物流、软件服务外包等现代服务业加速突破，电子商务交易额预计增长32.5%。由我市制定的《海洋牧场休闲服务规范》国家标准获批发布，建成国家级海洋牧场示范区5处、省级海洋牧场示范项目19处、省级以上休闲渔业示范基地33处，入选国家海洋经济创新发展示范城市。

　　重点项目建设取得新突破。实施市级重点项目185项、重点工业投资项目118项，谋划储备新旧动能转换重大项目116个。开展智能制造专项行动，威达机械、华力电机获批国家智能制造专项，天润曲轴、迪尚集团入选国家智能制造试点示范项目。招大引强取得新突破，马夸特开关、康得碳谷科技等一批重大项目签约落地，预计全市实际到账外资88亿元，增长10%。惠普公司入驻威海，亿和精密加工、大振电子等总投资12亿美元的5个配套项目先后落户，向打造全球打印机新高地迈出坚实步伐。

　　重点平台打造取得新突破。采取“一中心三平台”模式，启动创建国家区域创新中心。工信部电子信息技术综合研究中心、哈工大创新创业园等重大创新平台建设加快推进。设立树枝状及超支化聚合物应用创新中心等高端研发平台，助力前沿产业破土萌发。威高集团、迪尚集团获批国家级工业设计中心。服务贸易创新发展综合服务平台、国际海洋商品交易中心等大宗商品交易平台、文登西洋参交易中心等特色综合服务平台投入运行。

　　（二）综合施策、标本兼治，全力打赢五场攻坚战。结合做好十九大安保维稳工作，着力营造安全稳定和谐的社会环境。

　　安全生产攻坚战细致入微、扎实到位。组织开展安全生产大检查、百日攻坚治理等整治行动，排查治理隐患4.9万项。全力推进化工产业安全生产转型升级，全面加强海上安全管理，清理取缔海洋涉渔“三无”船舶1258艘。安全生产形势总体稳定，生产安全事故起数下降29.4%。

　　生态环保攻坚战多管齐下、集中整治。以中央环保督察、国家海洋督察为契机，深入开展环境保护突出问题排查整治。大气方面精准发力，狠抓扬尘治理，全面取缔整治1234家“散乱污”企业，完成53台电力企业燃煤锅炉超低排放改造，淘汰治理98%的分散燃煤锅炉。水环境方面河海统筹，504条河流全部推行“河长制”，完成城乡河道综合整治214公里，禁养区内513家畜禽养殖场全部关闭搬迁。扎实做好自然保护区问题整改，推进金海滩、大乳山等7个海岸带修复整治项目。荣成市成功创建全国首批生态文明建设示范市。

　　脱贫攻坚战聚焦短板、精准施策。推进贫困村整体提升工程，新建扩建扶贫产业项目92个，规划建设精准扶贫产业示范区15处、项目示范基地73个。推进精准扶贫直通车工程和金融扶贫工程，发放“富民农户贷”“富民生产贷”2.5亿元。规范扶贫资金管理使用，推动阳光扶贫、廉洁扶贫。

　　金融风险防控攻坚战分类处置、有效防范。开展金融风险排查处置专项行动、防范和打击非法集资活动、互联网金融风险专项整治，积极推动“破圈断链”，排查担保圈74个，防范化解171家风险企业和潜在风险企业的资金链风险，非法集资案发数量和金额分别下降63.6%和90.3%。

　　社会稳定攻坚战全民参与、共治共享。加强和改进信访维稳工作，开展化解信访积案集中攻坚活动。开展城乡社区协商示范点建设，推行警务助理、法律顾问进村进社区。海上平安建设经验成为全国典型，乳山市被评为全国平安建设先进县。

　　（三）聚焦重点、把握关键，着力增强三大动力。全面总结地级威海市成立30周年的经验，聚焦改革、开放、创新三大动力，千方百计提升发展活力和区域竞争力。

　　改革全面发力、多点突破。新争取21项省级以上改革试点，229项重点改革任务取得阶段性成果。362家规模企业完成规范化公司制改制，光威复材在创业板上市，新增新三板挂牌企业9家、区域股权市场挂牌企业25家。459个村完成集体产权制度改革，新增土地流转面积7万亩,乳山市获批全国农村综合性改革试点试验县。基础教育、学前教育省级改革试验区建设加快推进。开展生育保险和职工基本医疗保险合并国家试点。医药卫生体制改革、健康城市建设扎实推进，被确定为首批公立医院综合改革国家级示范城市。威海市及荣成市荣获首批全国社会信用体系建设示范城市。

　　开放先行先试、深度拓展。扎实推进中韩自贸区地方经济合作示范区、国家服务贸易创新发展、跨境电商综合试验区等试点工作，外贸进出口总额预计1380亿元，增长16%。服务贸易支行等多项服务贸易试点经验全国推广，服务贸易进出口额增长20%。开通国际海运快件业务，跨境电商零售出口增长1.6倍。主动参与“一带一路”建设，建设多式联运铁路枢纽中心，开通中欧集装箱班列，在沿线国家投资项目49个，贸易额增长44.8%。威海综合保税区建设进展顺利。乳山口港获批国家一类开放口岸。

　　创新要素集聚、活力增强。出台企业研发财政补助政策。促成产学研合作项目120项，技术交易额增长11.1%。引进山东铝业职业学院，与20家高校科研院所建立产学研合作战略联盟关系，成立威海职业教育联盟。完善“英创会”招才引智长效机制，实施国外智力引进项目49个，引进各类人才2.3万人。新增泰山产业领军人才23名，评选首届“威海工匠”47名，新增高技能人才8871人。获批创建全国质量强市示范城市，成功创建钓具产品国家产品质量提升示范区，威高集团获得中国质量奖提名奖，新增山东名牌产品27个。新建创业孵化基地10处，高区、迪尚集团入选国家“双创”示范基地。新增高新技术企业73家、科技型中小企业483家，各类市场主体总量增长14.4%。

　　（四）全域统筹、市域一体，加快推进新型城市化建设。坚持区域协同、城乡一体、陆海并重，持续推动全域城市化、市域一体化发展。

　　产城融合发展步伐加快。深入推进“四个突破”战略，东部滨海新城招商引资快速突破，全年签约入驻20个产业项目，市委党校、市职业中等专业学校、廉政教育基地投入使用，龙山湖主体工程完工；文登同城化、乳山产业结构调整步伐加快，南海新区获批省级经济开发区体制机制创新试点。双岛湾科技城、好运角旅游度假区、里口山城市山地公园等重点区域实施基础设施和产业项目127个，完成投资191.1亿元，增长9.1%。

　　人口市民化步伐加快。制定人口市民化规划和实施意见，加快推进本地农村居民市民化。实施38个老旧小区综合整治，开工棚户区改造1.9万户，新建新型社区36个。实施农村改厕9.6万户，农村改厕治污经验全国推广。虎山镇被评为全国特色小镇，羊亭镇、初村镇被评为省级特色小镇。

　　基础设施建设步伐加快。实施20项区域内应急供水工程，帮助267个因旱缺水村应急打井，充分发挥引黄调水工程作用应急调水，确保大旱之年城乡供水正常。加快推进乳山河地下水库、南海挡潮闸等水利工程建设，抗旱保障能力进一步增强。实施环山快速路、文莱高速公路等23项重大交通项目，香水河大桥建成通车，威海机场旅客吞吐量突破200万人次。新开工地下综合管廊18.5公里、海绵城市工程12平方公里。全面完成铁路沿线环境综合治理。整治城市违法建设7539处、429.8万平方米。被确定为全国“城市双修”试点城市和全省城市设计试点城市。

　　（五）完善制度、补齐短板，织密扎牢民生保障网。坚持从群众关心的问题抓起，全面提高民生保障和公共服务水平。

　　社会保障救助更加有力。实施职工大病保险制度，建立困境儿童基本生活保障制度，居民基础养老金标准、居民医保财政补助标准、失业保险金标准、城乡低保标准、特困人员供养标准都有新提高。建立政府救助保险制度，政府买单为全体市民投保重大自然灾害和意外事故保险。

　　公共服务供给更加到位。贴近群众需求办理28件民生实事。新建、改扩建中小学校18所，增加学位2万个。新建、改扩建公办幼儿园17所，增加学位4890个，普惠性幼儿园比例由80%提高到84.3%。设立医养结合机构16处，新增护理型养老床位1638张。组建医疗联合体16处，推动优质医疗资源共享和下沉。“双安双创”全域推进，成功创建国家食品安全示范城市和国家农产品质量安全市。

　　群众文化生活更加活跃。基层综合文化服务中心实现全覆盖，获批全国公共文化服务标准化试点城市。建成11家城市书房，为全民阅读创造便利条件。举办首届文化惠民消费季，发放文化惠民消费券2.8万张。举办铁人三项世界杯赛、威海市第五届运动会等赛事活动，新建改造健康主题公园12处、健康步道30条、镇级全民健身中心34处。

　　各项社会事业全面进步。制定普法责任清单，镇级公共法律服务中心实现全覆盖。国防动员、人民防空、民兵预备役工作进一步加强。民族、宗教、妇女儿童、外事、侨务、对台、档案、史志、地震、气象、慈善、残疾人等事业取得新进步。

　　（六）依法行政、从严治政，全力打造精简高效政务生态。扎实推进廉洁威海建设，进一步深化“放管服”改革，加快建设法治政府、服务型政府、廉洁政府。

　　便民利企服务进一步优化。实施政府提速工程，开展联系服务企业行动，着力让企业和群众办事更加省心、省力、省钱。取消、调整、减免4项政府性基金和15项行政事业性收费，进一步降低企业负担。实施“多证合一”“一照一码”，扩大“县域通办”业务事项范围，方便企业就近登记。开展减证便民专项行动，取消调整59项证明和盖章类材料。构建全项进驻、全域覆盖、全网统办的“三全”政务服务网络，率先完成“一窗式”“一门式”政务服务模式改革，完成市县镇联审联批国家服务标准化试点工作；建成全市统一的电子证照库，90%以上行政许可事项可全程网办，实现“零跑腿”或“只跑一次”。

　　行政权力运行进一步规范。配合市人大常委会开展地方立法工作，《威海市节约用水条例》《威海市饮用水水源地保护条例》等地方性法规正式颁布实施。坚持能减则减、宜放尽放，完善清单管理模式，编制公共服务事项目录、服务指南，削减市级行政权力事项88项。深入推进政务公开。推开综合行政执法体制改革和行政执法监督体制改革，实现“双随机一公开”监管全覆盖。

　　政府工作作风进一步改进。主动接受人大及其常委会法律监督和工作监督、政协民主监督、社会监督和舆论监督，办理人大代表建议63件、政协提案296件。驰而不息纠治“四风”，毫不松懈整治庸政懒政怠政，全年行政问责81人，查处发生在群众身边的不正之风和腐败问题255起，处理294人。

　　各位代表！过去一年的成绩来之不易，全市人民付出艰辛努力，社会各界给予大力支持。在此，我代表市人民政府，向全市各族人民，向人大代表和政协委员，向各民主党派、工商联和无党派人士、各人民团体，向驻威人民解放军指战员、武警官兵，向各位老领导、老同志，向关心支持威海发展的各界人士和海内外朋友，致以崇高敬意和衷心感谢！

　　在总结成绩的同时，我们也清醒地看到，我们的工作还存在许多不足，发展不平衡不充分的一些突出问题尚未解决，前进道路上面临不少困难和挑战。新动能培育不足，实体经济的发展活力有待增强；创新资源聚集能力不足，科技和人才短板仍是制约发展的重要瓶颈；城市国际化水平不高，招商引资和招才引智都亟待突破；交通、水利等基础设施建设还需进一步加强；海岸线、林地等自然资源管理存在薄弱环节；民生领域还有不少短板；安全生产、社会稳定、风险防控等方面的矛盾问题不容忽视；“放管服”改革还存在不到位的问题，隐形变异“四风”问题依然存在，政府系统廉政建设有待加强。对此，我们一定强化目标导向和问题导向，不回避矛盾，不推卸责任，从解决问题入手推动各项工作，努力让政府工作更加扎实、更富实效。

　　二、2018年政府工作的目标思路

　　党的十九大站在中国特色社会主义进入新时代的历史方位，确立了决胜全面建成小康社会、开启全面建设社会主义现代化国家新征程的目标。省第十一次党代会牢牢把握走在前列的目标定位，绘就山东由大到强战略性转变的新愿景。市第十五次党代会牢牢把握争当全省走在前列排头兵的目标定位，吹响实现现代化幸福威海建设新跨越的号角。我们要紧扣这些新目标、新愿景，紧紧围绕人民群众对美好生活的新需要、新向往，扎扎实实做好政府各项工作，坚持一张蓝图绘到底，努力把目标变为现实。

　　2018年是贯彻落实党的十九大精神的开局之年，是改革开放40周年，是决胜全面建成小康社会、实施“十三五”规划承上启下的关键一年。政府工作的总体要求是：全面贯彻落实党的十九大精神，以习近平新时代中国特色社会主义思想为指导，深入贯彻习近平总书记视察山东重要讲话、重要指示批示精神，坚持稳中求进工作总基调，坚持新发展理念，紧扣我国社会主要矛盾变化，按照高质量发展的要求，统筹推进“五位一体”总体布局和协调推进“四个全面”战略布局，牢牢把握争当全省走在前列排头兵的目标定位，以供给侧结构性改革为主线，以新旧动能转换重大工程为统领，大力实施全域城市化、市域一体化战略，大力实施产业强市、工业带动、突破发展服务业战略，大力实施城市国际化战略，统筹推进稳增长、促改革、调结构、惠民生、防风险各项工作，推动质量变革、效率变革、动力变革，促进经济平稳健康发展和民生福祉持续改善，努力实现现代化幸福威海建设新跨越。

　　主要预期目标为：生产总值增长8%左右，一般公共预算收入增长9%左右，外贸进出口稳定增长，城镇和农村居民人均可支配收入均增长8.5%左右，居民消费价格涨幅控制在3%左右，城镇登记失业率控制在2%以内，全面完成省下达的年度节能减排降碳约束性目标。

　　各位代表！党的十九大要求“创新引领率先实现东部地区优化发展”，中央经济工作会强调“支持东部地区率先推动高质量发展”，我们要牢牢把握争当全省走在前列排头兵的目标定位，在新时代历史坐标系中找准奋斗方位，主动对标先进，积极争先进位，按照“1+33333”的工作体系，制定实施三年行动计划，明确现代化幸福威海建设的路线图和时间表，努力打造更具影响力的宜居、宜业、宜游、宜学的卓越城市。

　　一是强化“一个统领”，坚定不移推动高质量发展。把新旧动能转换作为统领经济工作的重大工程，坚持产业引领、项目拉动、企业支撑，强化改革、开放、创新三大动力，加快建设现代化经济体系，促进新动能持续快速成长，推动全市经济在实现高质量发展上不断取得新进展。

　　二是实施“三大战略”，高点定位引领城市转型跨越。大力实施全域城市化、市域一体化战略，坚定不移地走特色城市化发展道路；大力实施产业强市、工业带动、突破发展服务业战略，夯实城市发展的产业根基、动力支撑；大力实施城市国际化战略，培育国际竞争新优势，拓展对外合作新空间，塑造城市开放新形象，丰富卓越城市的品质内涵，推动开放城市向国际化城市迈进。

　　三是突出“三个力保”，补齐短板夯实长远发展基础。坚持立足大局、着眼长远、把握关键，全力保证交通快速通达，全力保障水资源安全，全力保护海岸线等生态资源，谋划推动一批牵动全局的重要事项、重大工程，有效破除城市发展的瓶颈制约，进一步提升城市承载能力和发展后劲。

　　四是打好“三大攻坚战”，牢牢守住发展的底线红线。树立底线思维，强化抓重点、补短板、强弱项的责任担当，全力以赴打好防范化解重大风险、精准脱贫、污染防治的攻坚战，坚决保住基本、兜住底线，着力营造安全稳定的社会环境。

　　五是坚持“三个聚焦”，精准发力推进重点工作加速突破。坚持聚焦区域、聚焦产业、聚焦政策，找准区域发展的突破口，提高产业发展的精准度，打好政策支持的组合拳，强化谋篇布局、顶层设计，增强工作的前瞻性、战略性、全局性，推动重点区域率先突破、重点产业快速发展、重点工作加速见效。

　　六是强化“三个整合”，高效联动保证工作落实。加快整合12345政务服务热线、整合共享政务信息系统、整合政务督查力量，充分运用大数据等信息化手段，精准了解社情民意，高效办理群众诉求，全面提升政府决策和服务水平，进一步增强执行力，雷厉风行狠抓落实，更好服务群众、服务企业、服务发展。

　　各位代表！追求卓越、争创一流，是地级威海市成立30年以来威海人民积攒的精气神，也是我们迈入新时代、开启新征程的动力之源。我们要不忘初心、继续前进，时刻保持不进则退的危机意识，确立更高发展定位，树立更高发展标杆，奋力推动创新发展、持续发展、领先发展。

　　三、2018年政府工作重点

　　（一）牢牢把握主线、紧扣动能转换，加快建设现代化经济体系。坚持以供给侧结构性改革为主线，突出制造业优化升级，在“破”“立”“降”上下功夫，大力破除无效供给，大力培育新动能，大力降低实体经济成本，提高全要素生产率。

　　实施重点产业集群培育工程。突出龙头带动、链条延伸、要素聚集，实施“中国制造2025”威海行动计划，集中力量培育七大千亿级产业集群。新一代信息技术产业集群，推动实施惠普打印机、亿和精密加工等重点项目，规划布局集成电路产业，打造计算机外设产品研发生产基地。新医药与医疗器械产业集群，推进实施威高手术设备和可降解支架等重点项目，争取在高精尖医疗设备、康复医疗设备等领域形成集聚效应。先进装备与智能制造产业集群，重点发展高档数控机床、医用特种机器人、轨道交通装备、节能环保装备、高技术船舶与海洋工程装备等优势产品。碳纤维等复合材料产业集群，推动康得碳谷科技、拓展纤维航空复合材料等重点项目建设，延伸碳纤维综合制品产业化链条，加快发展先进高分子、石墨烯等前沿材料。海洋生物与健康食品产业集群，重点发展海洋保健品药品、海洋功能食品等高附加值产品，进一步提高中国海洋食品名城的国际知名度。时尚与休闲运动产品产业集群，推动纺织服装、家纺地毯、皮具钓具等传统产业与文化时尚元素、工艺设计跨界融合，打造全国知名的时尚创意城市和时尚产业基地。康养旅游产业集群，重点引进高端医疗保健、养生疗养、健康管理、医学美容等健康服务机构，加快建设国际医疗健康产业城、健康医疗大数据中心和康养特色小镇。

　　实施新经济培育工程。坚持以“四新”促“四化”，聚焦数字经济，实施工业互联网战略和大数据战略，促进物联网、云计算、大数据、人工智能与实体经济深度融合，推进企业商业模式变革和流程再造，加快在休闲经济、共享经济、创意经济、网络经济等领域培育新动能。推动“工业制造”向“工业智造”转变，实施机器换人、数字化与智能工厂建设、智能制造示范提升“三大工程”，引导企业探索应用各类智能制造新模式。推动“生产型制造”向“服务型制造”跨越，支持推广总集成总承包、个性化定制、柔性化生产等商业模式，鼓励企业由单一产品供应商向系统解决方案提供商转变。推动“单一业态”向“多元业态”融合，促进“文化+”“物流+”“旅游+”“健康+”“养老+”等跨界融合渗透，引导企业拓展发展空间。推动“互联网+”向“云+”迈进，实施“企业上云”专项行动，引导企业利用大数据和云计算，实现管理、业务等方面的数据化转型，插上在“云端”飞翔的翅膀。

　　实施现代服务业突破工程。坚持发挥优势、补齐短板、突破重点，大力推动“六个提升”，促进传统服务业优化提升，新兴服务业加速成长。提升游客体验丰富性，围绕争创国家全域旅游示范区，推进黄金海岸提质、乡村旅游提升、景区旅游提标，开发工业旅游、红色旅游、冬季旅游、夜间旅游等系列产品，构建业态丰富、一体发展的全域旅游格局，让游客充分感受城市的品质和温度。提升康养品牌吸引力，积极培育国医康养、海洋康养、温泉康养等新兴业态，构建全领域、全周期、全链条康养产业体系，打响“四季威海·康养福地”品牌。提升现代物流通畅度，推动港口资源整合，促进国家一类开放口岸突出特色、加快发展，加大“无水港”合作建设力度，建设多式联运铁路枢纽中心，大力发展临港产业园区、铁路物流产业园区，做强做优跨境物流、冷链物流等产业，创建冷链物流示范城市。提升商贸流通特色化，优化特色商圈布局，大力发展电子商务，推动传统商贸和实体商业转变经营模式，培育“商品+服务”“购物+休闲”“消费+体验”等新业态。提升商务服务专业化，积极培育中介服务机构，强化精准招商提升酒店业发展层次，增强会展活动承接能力。提升生活服务便利性，加快发展家政服务、教育培训、体育休闲等产业，更好满足群众多层次、多样化消费需求。

　　实施海洋经济转型突破工程。强化科学用海、科技兴海、产业强海、生态护海“四个导向”，深入推进蓝色经济区建设，争创国家海洋经济发展示范区，推动海洋渔业大市向海洋经济强市跨越。聚焦海洋产业转型，突破发展海洋生物制药、海洋健康食品、海洋高端装备等产业；加强国家级远洋渔业综合基地、海洋牧场示范区建设，减船转产压减近海捕捞产能1.8万千瓦。聚焦产业链条延伸，创新海洋经济融合发展新模式，加快培育海工设计、海洋物流、涉海服务等新兴产业。聚焦综合平台建设，加快建设海洋科技产业聚集示范区，打造有影响力的海洋科技创新中心；支持海洋高新技术产业园创建国家农业高新技术产业示范区，推动15个海洋特色园区创新发展；加快国家浅海综合试验场、海洋生物遗传育种中心、山东船舶技术研究院、迪沙集团海洋药物与功能食品研发中心等平台建设，提高海洋科技成果转化能力。聚焦金融服务创新，组建海洋投资公司，创新海上保险、航运融资、大宗海产品交易等海洋金融服务，引导社会资本投资海洋经济。聚焦优势品牌培育，打响优质海产品、休闲渔业等区域品牌，加快有定价权的海产品综合服务平台建设，支持企业掌握行业主导权。

　　实施高效生态安全农业提升工程。坚持质量兴农、绿色兴农，推动农业政策从增产导向转向提质导向，调好调顺调优农业结构，加快培育农业发展新动能。大力发展高效农业，建设西洋参、无花果、大姜、茶叶、葡萄等特色种植区，新增现代果园2万亩；培植壮大农业“新六产”，高标准建设汪疃农耕文化体验园，打造山水田园示范区。大力发展生态农业，开展绿色防控技术推广行动，实施耕地质量提升计划和优质粮食工程，新增水肥一体化面积7万亩，强化技术创新保障粮食安全。大力发展安全农业，加强追溯体系和信息平台建设，增加绿色优质农产品供给。

　　实施质量品牌提升工程。弘扬“幸福威海·品质承载”城市质量精神，支持企业在增品种、提品质、创品牌上攻坚发力，争创全国知名品牌创建示范区，创建全国质量强市示范城市。实施品牌强农工程，加强地理标志品牌保护，擦亮“威海农产·品味自然”“威海苹果·甜蜜诱惑”等农产品区域公用品牌形象。开展重点行业质量提升行动和群众性质量管理活动，夯实企业质量管理基础。推进国家碳纤维及其复合材料产业计量测试中心等公共检验检测服务平台建设。大力实施标准化战略，争创国家医疗器械技术标准创新基地，鼓励更多企业争当标准“领跑者”。

　　各位代表！加快新旧动能转换，建设现代化经济体系，相关工作都要工程化、项目化，围绕产业来谋划，依托项目来推进，依靠企业来实施。今年全市重点推进116项新旧动能转换重大项目、155项市级重点项目、147项市级重点工业投资项目。要按照“五个牢牢抓在手上”的思路，把目标和责任落实牢牢抓在手上，把产业和项目培育牢牢抓在手上，把园区和平台建设牢牢抓在手上，把政策和机制创新牢牢抓在手上，把环境和能力建设牢牢抓在手上，进一步夯实经济转型升级的基础，推动经济发展质量变革、效率变革、动力变革。

　　（二）强化创新驱动、补齐人才短板，加快建设创新型城市。围绕创建国家创新型城市，争创国家促进科技和金融结合试点城市、国家知识产权示范城市，加快实施创新驱动发展战略。

　　加强创新平台建设。加快创建国家区域创新中心，支持工信部电子信息技术综合研究中心、哈工大创新创业园建设，在高性能医疗器械领域争创国家制造业创新中心，引进更多协同创新的平台机构，构建“1+3+N”创新格局。开展企业研发平台提质升级行动，依托行业优势企业争创国家技术创新中心，支持更多企业创建省级以上技术研发平台，促进创新平台开放联合、资源共享。建设军民科技协同创新平台，打造军民融合创新示范区。

　　加强创新产业园区建设。加快国家自主创新示范区建设，推动医疗器械与生物医药、电子信息与智能制造、碳纤维等高端产业园区加快布局创新产业项目，构建“一区多园”创新产业发展格局。建设核能利用国际创新示范园，加强核能相关产业开发利用。规划布局中欧水处理及膜技术创新产业园。加强校地协同创新，吸引高校院所来我市设立产业研究院和科技园、创新园。

　　加强创新型企业培育。强化以企业为主体的创新体系建设，实施“千帆计划”“科技型中小企业雏鹰成长行动”，培育一批具有创新能力的排头兵企业。开展单项冠军企业培育提升行动，支持企业走“专精特新”发展道路，培育更多的瞪羚企业、独角兽企业和隐形冠军企业。加强国家“双创”示范基地建设，搭建国家级服装设计、建筑设计创新创业平台，构建全链条“双创”生态圈，促进大众创业、万众创新上水平，打造充满活力的“最暖创业城市”。

　　加强创新人才引进培养。深化人才发展体制机制改革，完善招才引智政策。深化提升“威海英才计划”，制定离岸创新人才、青年创新人才支持政策，实施干部国际化能力培养“双百计划”，升级“产学研合作特聘专家”“校地合作人才”等人才工程，打响“英创会”引才品牌，加强与欧美同学会等团体联谊交流，大力引进国家“千人计划”、泰山系列人才工程专家团队等创新人才。密切与驻威高校共建合作，促进更多高校毕业生留威创业就业。深化产教融合，引导职业院校开门办学，鼓励企业参与举办职业教育，培育更多适应产业需求的高技能、创新型“威海工匠”。

　　各位代表！建设创新型城市，需要整合各方创新力量，集聚各类创新资源。要加快构建政、产、学、研、用、金、服“北斗七星”一体联动的创新模式，促进创新与产业融合、研发与需求对接、科技与金融互动，努力形成创新要素流动、整合和集聚的最优生态，让威海成为产学研合作的密集区、创新产业发展的新高地。

　　（三）坚持高点定位、对接国际标准，推动形成全面开放新格局。全面总结改革开放40周年的经验，牢牢抓住城市国际化战略、招商引资、开放型经济新体制“三个着力点”，进一步拓展开放的范围和层次，在新一轮高水平对外开放中增创新优势。

　　高标准实施城市国际化战略。坚持世界眼光、国际标准、本土优势，制定城市国际化建设行动纲要，建立评价指标体系，推动建设开放型国际经济活跃城市、区域性国际交往门户城市、标准化国际生态宜居城市。加强城市功能国际化、规范化建设，加快双语标识、国际学校、涉外门诊、自助导游等服务体系建设，在城市建设、语言环境、文化教育、医疗服务、休闲旅游、社会管理等方面与国际化标准全面接轨。开展系列国际交流活动，推进友好城市交流、海外联络联谊，提高城市知名度和国际影响力。

　　高效能推进精准招商。坚持把招商引资作为经济工作生命线，全面优化外商投资环境，运用整体思维谋划产业招商，瞄准世界500强、央企百强企业及行业隐形冠军企业，有针对性地开展建链招商、补链招商、强链招商。探索组建企业化管理、市场化运行的招商机构，提升招商队伍专业化水平。强化重点区域合作，着力深化欧美、深耕周边，争取对德国制造业招商取得新突破，争创中德中小企业合作区。坚持大胆假设、小心求证，统筹算好眼前和长远、静态和动态、局部和全局的平衡账，增强招商引资的精准度和实效性。

　　高水平构建开放型经济新体制。务实推进中韩自贸区地方经济合作示范区建设。扎实开展国家服务贸易创新发展试点，培育服务出口基地和特色小镇，设立“一带一路”国家云服务运营中心，加快建设服务贸易产业园。推动威海综合保税区正式封关验收，加快发展保税加工、保税物流、保税服务产业。加强国际贸易“单一窗口”建设和推广，提高对外贸易便利化水平。推行行政“二号章”、“大部制”、用人制度、薪酬制度等改革措施，创新开发区体制机制。发挥高区、经区、临港区等国家级开发区的主阵地作用，推动开发区“二次创业”。

　　高层次深化双向开放合作。坚持“引进来”和“走出去”相结合，主动融入“一带一路”建设。积极培育本土跨国公司，鼓励企业创新对外投资方式，并购境外研发、设计、营销、品牌等价值链优质资源，设立境外协同创新中心、工业设计中心、品牌营销中心，以投资带动贸易发展、产业发展。实施境外百展市场开拓计划，提升国际食品博览会、国际渔具博览会等展会层次。坚持扩大出口与高质进口并重，进一步优化外贸结构。积极培育外贸新业态，争创国家级跨境电商综合试验区。

　　（四）全面深化改革、优化发展环境，激发各类市场主体活力。坚持使市场在资源配置中起决定性作用，更好发挥政府作用，实施“改革落实行动计划”，加快推进基础性关键领域改革，提高各项政策的精准性、靶向性。

　　优化政策支持环境。做好政策法规立改废工作，建好政策“工具箱”，形成支持实体经济发展的政策叠加效应。全面落实支持国有企业、民营企业发展的政策，让企业充分享受政策红利。降低实体经济成本，降低制度性交易成本，全面清理涉企收费，组织开展“红顶中介”专项治理。帮助企业降低用能、物流成本。推进财政事权和支出责任划分改革，调整优化财政支出结构，压缩一般性支出，深入推进“拨改投”，加大对重点领域和项目的支持力度。健全企业家参与涉企政策制定机制，避免出现“桃子政策”“抽屉政策”，实实在在增加企业的获得感。

　　优化金融服务环境。大力发展普惠金融，强化科技支行、小微企业贷款风险补偿基金、小额贷款保证保险、农业融资担保等政策措施，帮助企业破解融资难题。推动威海蓝海银行和城商行、农商行等地方法人银行机构完善治理结构。推动企业加快对接多层次资本市场，争取新增上市挂牌企业15家。鼓励企业提高直接融资比重，促进更多金融活水流向实体经济。

　　优化投融资环境。强化实体经济吸引力和竞争力，优化存量资源配置，促进有效投资特别是民间投资合理增长。整合投融资公司，加强政府投资引导基金运营管理，推动设立产业发展基金，助力新兴产业和创新型企业加速成长。加强政府债务管理，严格控制新增债务。深化国企国资改革，开展混合所有制改革试点，推动国有民营经济双向参股。规范PPP项目运作，引导更多社会资本参与基础设施、公共服务等领域建设。

　　优化市场监管服务环境。正确处理管理与服务的关系，健全涉企服务和市场监管机制。持续深化商事制度改革和投资审批制度改革，取消不必要的行业门槛和隐性壁垒。规范涉企检查行为，推行跨部门“随机联查”，避免干扰企业正常生产经营。探索包容创新的审慎监管机制，建立事中事后监管新模式。

　　优化社会信用环境。深入开展信用提升年活动，加强政务诚信建设，引领商务诚信、社会诚信和司法公信建设走向深入。深化统计管理体制改革，加强统计数据质量管理。推进部门和行业信用管理，探索实施信用积分评价制度，扩大市场主体信用记录覆盖面。强化行业自律和信用监管，实施联合激励惩戒，让守信者一路畅通、失信者寸步难行。

　　优化企业家成长环境。完善联系服务企业机制，加快构建亲清新型政商关系，营造尊重企业家价值、鼓励企业家创新、发挥企业家作用的良好社会氛围。实施优秀企业家培育计划，开展企业家“青蓝接力”行动，助力年轻一代企业家健康成长。

　　（五）坚持生态立市、推动绿色发展，提供更多优质生态产品。树立和践行绿水青山就是金山银山的理念，巩固提升生态环境比较优势，积极创建国家生态文明建设示范市。

　　坚持不懈倡导绿色发展方式。实施百企节能降耗工程和节能产品惠民工程，培育循环经济示范企业和示范项目，推广应用节能新技术，调整能源结构、运输结构。严格实行区域新建项目排污总量减量、等量替代，从源头上减少污染。开展全民节水行动，推进田间工程节水改造，提高工业用水重复利用率。开展节约型机关、绿色家庭、绿色学校创建活动，推动形成绿色生活方式。

　　全力以赴解决环境突出问题。加大中央环保督察反馈问题整改力度，对中央环保督察交办的245件案件组织回头看。结合即将开展的省环保督察，继续开展环境突出问题综合整治攻坚行动。大气污染防治抓“稳固”，编制大气污染源清单，重点抓好“散乱污”企业和分散燃煤锅炉整治，强化扬尘污染防治，确保环境空气质量继续稳定达到国家二级标准。水污染治理抓“提升”，扎实推进工业废水污染源全面达标、工业集聚区水污染集中治理、城镇生活污染防治、流域环境和近岸海域综合治理等工作，全力开展集中式饮用水水源地环保执法行动。土壤污染治理抓“基础”，开展土壤环境质量监测，加强农业面源污染防治，建成固体废物处置中心，提高危险废物规范化管理水平。

　　多措并举保护生态系统。培育一批专门从事生态保护修复的专业化企业，高标准实施生态系统保护和修复工程。实施河道水库绿化整治工程，打造沿河环库的绿色林带、景观林带和经济林带。完善城市山体公园管理机制。支持威海植物园建设。坚持绿化、花化、彩化统筹兼顾，年内成片造林6万亩，推动“绿满威海、四季多彩”。全面落实“河长制”，开展“清河清库”行动。探索推行“湾长制”，开展黄金海岸生态保护试点，严控围填海项目，推动近岸海域沙滩养护、还滩还海，恢复海岸自然属性。

　　夯实责任加强生态环境监管。建立“一个系统、四个平台”，完善环境问题处置长效机制，进一步健全自然资源资产产权制度，建立生态补偿机制。实施环保机构监测监察执法垂直管理制度改革。加快建设河流跨境断面、污水溢流口自动监测监控设施，新建20个环境空气自动监测站。开展第二次全国污染源普查。全面推行排污许可证制度，加强企业环境信用体系建设。

　　各位代表！生态环境是威海最宝贵的财富，必须像对待生命一样来对待。要正确看待我市生态环境比较优势和环保工作中的薄弱环节，进一步完善专业监管与“啄木鸟”式监管相结合的环境监管机制，把山、海、河、林、湿地等各类生态资源守护好、利用好，让碧海蓝天、绿水青山成为威海最靓丽的城市名片。

　　（六）坚持全域统筹、强化规划引领，加快推动市域一体化发展。坚持以人的城市化为核心，以全域城市化、市域一体化为方向，加快同城融合、协同发展。

　　统筹推进“多规合一”。树立“全域威海观”，编制市域一体空间统筹规划，统筹全域生态、生产、生活空间，推动市域统筹一体化布局、产城融合一体化发展、城乡均衡一体化提升、陆海并重一体化开发、基础设施一体化建设、生态环境一体化保护。启动新一轮城市总体规划编制，划定生态保护红线、永久基本农田、城镇开发边界三条控制线。统筹国民经济和社会发展规划、城乡规划、土地利用规划及生态保护、林地保护、海岸带保护、水安全保障等专项规划，建成“多规合一”综合应用系统，形成城乡统筹、全域覆盖、要素叠加“一张蓝图”。加强非城镇建设用地管理，集约节约利用土地资源。推动城市设计全覆盖和智能化，统筹建筑布局，协调城市景观，保持特色风貌。

　　统筹推进重点区域开发。深入实施“四个突破”战略，扎实推进国家产城融合示范区建设。集中力量开发建设东部滨海新城、双岛湾科技城、南海新区等重点区域，加快周边区域城市化改造，实施国际经贸交流中心、高端人才创新创业基地等重点项目，加速聚集产业项目。推动好运角旅游度假区、里口山城市山地公园、乳山滨海新区、昆嵛山旅游度假区等区域生态保护性开发，打造知名山海旅游目的地和“慢生活”体验区。

　　统筹实施“城市双修”工程。优化“城市家具”设计和布局，塑造整洁和谐的公共空间。制定户外广告设置规划导则，推行分区控制。加大城市违法建设治理力度，推进“城市伤疤”治理。实施19个老旧小区综合整治。完善物业管理机制，提高物业管理水平。推开新建住宅全装修。推进地下综合管廊和海绵城市建设。健全城市网格监管体系，提高城市精细化管理水平。完善5G通信网络等数字基础设施，加快推进智慧城市建设。

　　统筹实施乡村振兴战略。科学制定乡村振兴战略规划，健全城乡融合发展体制机制，加快推进农业农村现代化。加强农村综合产权交易中心建设，新增土地流转面积5万亩。积极培育新型农业经营主体，支持发展家庭农场，新增农业科技示范主体1000个。加快美丽乡村建设，实施“百村示范、千村提升”行动，推动建设田园综合体。开展新一轮乡村文明行动，夯实乡村治理基层基础。加快户籍制度改革落地步伐，完成500个村集体产权制度改革，推动本地农村居民市民化。坚持因地制宜、一镇一策，精心培育特色小镇、重点示范镇，打造一批乡村振兴战略示范村。

　　统筹推进基础设施建设。按照构建市域一体、国内通达和联通海外“三大交通圈”的思路，聚焦客流通达、物流集散，编制全市综合交通网发展规划。加快推进莱荣高铁、文莱高速公路、环山快速路、乳山口跨海大桥等重大项目，规划疏港铁路，完成城市轨道交通规划报批，做好威海新机场选址工作，全方位开拓空中航线，加密班列开行频次。围绕建立“南北连接、中心贯通、江黄共济”的水资源保障体系，统筹做好广蓄水、引客水、淡海水、用中水、治污水的文章。实施龙角山、坤龙邢水库增容和黄垒河拦蓄利用等工程，做好南水北调南线工程规划论证，加强中水利用管网规划建设。坚持陆海统筹、水岸共治、管护并重，加强海岸线等生态资源保护。实施“蓝色海湾”“生态岛礁”等工程，推进黄垒河、乳山河、母猪河分段综合整治，加强自然保护区和湿地、林地资源保护恢复工程建设。组织实施4大类、43项城建重点工程，加快“一带四轴”绿道网络建设。

　　（七）尊重群众意愿、增进民生福祉，切实提高保障和改善民生水平。紧紧围绕解决民生领域的不平衡不充分问题，积极主动回应群众关切，进一步加强基本公共服务和基本民生保障。

　　办好人民满意教育。坚持教育优先发展，扎实推进基础教育综合改革试验区等试点工作。实施学前教育三年行动计划，解决好婴幼儿照护和儿童早期教育服务问题。建立消除“大班额”长效机制，着力解决中小学生课外负担重等突出问题。推进立德树人工作，启动中小学图书馆式学校建设和师生“大阅读”工程，加强学生艺术素养评价。推进特色高中建设，提升高中阶段教育普及水平和质量。扩大中职学生接受高等教育规模。东部滨海新城优质高中投入使用，加快推进市中小学生综合实践教育中心三期工程。

　　加强就业和社会保障。注重解决结构性就业矛盾，解决好性别歧视、身份歧视问题。实施积极就业政策，推动公共就业服务、职业技能培训、援企稳岗政策延伸覆盖，城镇新增就业3.3万人。深入实施全民参保计划，启动实施职工长期护理保险制度，深化医保支付方式改革，提高居民医保财政补助标准。提高城乡最低保障标准、困境儿童生活保障标准。加快建立多主体供应、多渠道保障、租购并举的住房制度，完善促进房地产市场平稳健康发展的长效机制，支持发展住房租赁市场，新开工棚户区改造2.9万户，采取租补结合的方式，帮助住房困难家庭实现“安居梦”。

　　打赢脱贫攻坚战。坚持既不降低标准，也不吊高胃口，瞄准特定贫困群众精准帮扶，对全部建档立卡贫困户再摸底、再确认，将因病因灾新致贫人口纳入扶贫范围，“一户一策”细化帮扶措施，实施有效救助。因村制宜推进社会扶贫、金融扶贫、产业扶贫，打造扶贫示范基地100个。加强考核监督，完善长效机制，巩固提升脱贫成果，确保小康路上不让一户一人掉队。

　　加快健康城市建设。深化医药卫生体制改革，健全分级诊疗、现代医院管理和药品供应保障制度，建成紧密型医疗联合体6处，着力解决“看病难、看病贵”问题。加快国家中医药综合改革试验区建设，推动国医院开业运营。实施“健康细胞”工程，强化重大疾病预防控制，提升计划生育和妇幼健康服务水平。开展残疾人精准康复行动。广泛开展全民健身活动，举办铁人三项世界杯赛，做精做活帆船、垂钓等品牌赛事。推进国家医养结合试点、居家和社区养老服务改革试点，新建社区养老服务中心20处。

　　扎实推进文化建设。积极创建国家公共文化服务体系示范区。深入实施文化惠民工程，开展文化惠民消费季活动。推动“全民阅读·书香威海”建设，新建城市书房8处、共享图书柜25处。启用市图书馆新馆、群众艺术馆新馆。加强动漫创意、艺术品交易、油画创作等基地平台建设，积极培育新型文化业态。实施优秀传统文化传承弘扬工程。发掘老洋房、乡村记忆馆、文物保护遗址的文化内涵，讲好威海故事，留住威海记忆。深化新闻出版、广播影视等领域文化体制改革。积极培育和践行社会主义核心价值观，深化“君子之风·美德威海”建设，开展文明单位、文明家庭、文明校园等群众性精神文明创建活动，扎实开展“爱文明家园、做文明市民”行动，巩固提升文明城市创建成果。

　　各位代表！人民群众对美好生活的需要，就是政府工作的努力方向。在广泛征求群众意见建议的基础上，今年我们确定为群众办理12件实事：（1）新建改扩建中小学校26所、普惠性幼儿园17所，持续改善城乡办学条件；（2）推行家庭医生签约服务，城乡居民签约服务率分别达到30%和65%以上；（3）实施市区7条瓶颈路打通工程，治理“背街小巷”20处；（4）绿化整治河道35条，优化沿河生态环境；（5）改造农村供暖设施281处，实现农村公共场所清洁供暖全覆盖；（6）建设小游园23处、山体公园5处，新增城市绿道48.8公里，方便群众就近健身休闲；（7）推动“厕所革命”，改造提升中心城区公共厕所25处，完成农村改厕11.3万户；（8）中心城区开通优化7条公交线路，提高换乘便利性；（9）利用老建筑建设城市博物馆等公益文化空间8处，丰富群众文化体验；（10）新建社区居家养老服务组织72个，打造15分钟社区养老服务圈；（11）实施农村饮水安全巩固提升工程，改善252个村的居民饮水条件；（12）新建农业社会化综合服务中心31处，为农户提供一站式、全链条服务。我们将紧扣群众所思所想所盼，聚焦痛点难点堵点，坚持尽力而为、量力而行，一件事情接着一件事情办，一年接着一年干，努力把民生实事办到群众的心坎上，实实在在增加群众的获得感、幸福感。

　　（八）筑牢基层基础、凝聚攻坚合力，加强和创新社会治理。大力加强平安威海建设，打造全民共建共治共享的社会治理格局，全方位提高人民群众安全感。

　　全力夯实社会治理基石。坚持政府治理和社会调节、居民自治良性互动，强化城乡社区治理，选优配强“小巷总理”和村居工作队伍，高标准建设农村社区服务中心157处，建好用好四级综治中心。健全立体化社会治安防控体系，打好公安基层基础建设三年攻坚战，建设应急通信政务专网、移动警务应用平台。依法严厉打击传销、黄赌毒等违法犯罪活动。深入实施“七五”普法规划。健全公共法律服务体系。有效开展法律援助。深化社会组织管理制度改革，推进社会组织承接政府转移职能。

　　全力守住安全生产底线。健全风险分级管控和隐患排查治理双重预防机制，落实安全生产领域失信行为联合惩戒机制，强化企业主体责任落实。深入开展“大快严”集中行动、化工产业安全生产转型升级专项行动、道路交通“平安行·你我他”行动，加强重点领域专项治理。加强气象、水文、地震等工作，健全综合防灾减灾体系。强化应急教育培训、装备配备和预案演练，提高应急处置能力。开展“食安威海”品牌引领行动，实施餐饮质量安全提升工程，保障群众“舌尖上的安全”。

　　全力筑牢风险防控屏障。继续开展排查安全隐患防范四类风险专项行动。加强金融风险防控，做好企业担保圈、民间借贷、互联网金融等重点领域风险防范和处置，严厉打击违法违规金融活动，守住不发生系统性金融风险的底线。着力解决网上虚假信息诈骗、倒卖个人信息等突出问题。完善社会矛盾预防和排查化解综合机制，推进诉调对接中心、行业调委会、人民调解员队伍建设，推进律师参与矛盾化解、仲裁参与诉调对接、村级法律顾问兼任人民调解员工作创新，用心用情用力解决群众利益诉求。

　　协调发展各项社会事业。加强国防动员、民兵预备役、打击走私和人民防空建设，巩固和促进军政军民团结。依法保障妇女、儿童、老年人、残疾人合法权益，大力发展社会福利和公益慈善事业。继续做好民族、宗教、外事、侨务、对台、仲裁、档案、史志、对口支援和扶贫协作工作。

　　四、建设人民满意的服务型政府

　　各位代表！为人民服务、对人民负责、受人民监督、让人民满意，是政府的职责与使命所在。我们一定坚持以全面从严治党统领政府各项工作，更加奋发有为、勤勉务实，切实提升管理服务效能，努力使政府工作赢得人民群众的信任和支持。

　　（一）强化政治过硬、执行有力的责任担当。坚持把政治建设摆在首位，推进“两学一做”学习教育常态化制度化，开展“不忘初心、牢记使命”主题教育，强化“四个意识”，坚决维护以习近平同志为核心的党中央权威，自觉以习近平新时代中国特色社会主义思想武装头脑、指导实践、推动工作。坚持把雷厉风行、专业务实作为政府鲜明的工作风格，推行工作项目化、项目清单化、清单责任化，量化细化实化政府工作。完善决策部署执行、监督、考评、奖惩等工作机制，健全正向激励、容错纠错、澄清保护机制，推行跟踪督查、综合督查、下沉督查，落实主体责任，确保政令畅通。

　　（二）强化依法行政、阳光运行的法治理念。坚持把政府工作全面纳入法治轨道，落实好重大决策公众参与、风险评估、合法性审查等五项制度，强化行政决策法定程序的刚性约束。树立整体政府理念，深化大部门制度改革，优化部门内部科室设置，推动行政职能整合和资源优化配置。加强行政执法监督，促进严格规范公正文明执法。自觉接受人大法律监督、政协民主监督，认真办理人大代表建议和政协提案，广泛听取民主党派、工商联、无党派人士意见。深入推进政务公开，畅通政民互动渠道，让人民群众更直接更有效地监督政府。

　　（三）强化务实高效、便民利企的服务意识。持续深化“放管服”改革，深入推进削权减证等“五大行动”，创新政务服务体制机制，以更彻底的放权、更到位的监管、更精准的服务，为企业松绑、为群众解绊、为市场腾位。深入推进“互联网+政务服务”，深化政务服务平台建设与应用，推动线上线下深度融合，让数据多跑路、群众少跑腿。进一步整合公共资源交易平台，实现公共资源交易全流程电子化。推动“政府上云”，加快政务信息系统整合共享，建成全市共用的电子政务云。整合建立全市统一的12345政务服务热线，实行“一号受理、限期办结、综合督办、双向反馈、回访全覆盖”，确保群众诉求快速高效处置。

　　（四）强化清正廉洁、纪律严明的优良作风。深入推进廉洁威海建设，开展廉政教育和廉政文化建设，严格落实全面从严治党责任，把廉洁从政贯彻到政府工作各环节。推进审计监督全覆盖，加强对重点领域、关键环节、重要岗位权力运行的监督制约，深化扶贫领域腐败和作风突出问题专项治理，铲除滋生腐败土壤。持续整治“四风”问题，坚决反对特权思想和特权现象，巩固拓展落实中央八项规定精神成果。用好监督执纪“四种形态”，抓早抓小、防微杜渐，严厉查处侵害群众利益的“害群之马”，以反腐倡廉的决心和成效，取信于民、服务于民、造福于民。

　　各位代表！新思想引领新时代，新使命开启新征程。让我们紧密团结在以习近平同志为核心的党中央周围，在市委的坚强领导下，不忘初心、牢记使命，以更加饱满的激情、更加务实的作风，不断开创各项工作新局面，努力争当全省走在前列排头兵，为实现现代化幸福威海建设新跨越而努力奋斗！
